# The Expression of BNP, ET-1, and TGF-β1 in Myocardium of Rats with Ventricular Arrhythmias

**DOI:** 10.3390/ijms20235845

**Published:** 2019-11-21

**Authors:** Meihui Tian, Ying Xiao, Jiajia Xue, Yuan Zhang, Yuqing Jia, Xinyi Luo, Tianqi Wang, Baoli Zhu, Zhipeng Cao

**Affiliations:** Department of Forensic Pathology, School of Forensic Medicine, China Medical University, No. 77 Puhe Road, Shenyang North New Area, Shenyang 110122, Liaoning, China; tianmh0619@163.com (M.T.); xiaoying20191121@163.com (Y.X.); jjxue0218@163.com (J.X.); zhangyuan072010@sina.com (Y.Z.); jia214@hotmail.com (Y.J.); luoxinyi07@163.com (X.L.); tianqi_wang_wtq@163.com (T.W.)

**Keywords:** ventricular arrhythmia, SCD, BNP, ET-1, TGF-β1

## Abstract

Ventricular arrhythmia (VA) is a major component of sudden cardiac death (SCD). To investigate the expression of brain natriuretic peptide (BNP), endothelin-1 (ET-1), and transforming growth factor-beta 1 (TGF-β1) during VA, we established a rat model of VA induced by BaCl_2_ solution through a microinjector pump. PD142893 (ET-1 receptor blocker) and SB431542 (TGF-β1 receptor type I blocker) were used to explore the effect of ET-1 and TGF-β1 on BNP expression in the myocardium after VA. BNP, ET-1, and TGF-β1 in rat myocardium were assayed by western blot and immunohistochemical staining for proteins, and real-time quantitative polymerase chain reaction for mRNAs. We found increased expression of BNP and ET-1 in rat myocardium that was associated with the duration of VA. However, TGF-β1 protein expression remained unchanged. Such early increases in BNP and ET-1 may be attributed to fatal arrhythmias associated with SCD, suggesting these may be novel biomarkers of this disease. After intraperitoneal injection of PD142893 and SB431542, respectively, BNP was downregulated in the myocardium of the left ventricle; however, this was abrogated by co-application of the two inhibitors. These results suggested that both ET-1 and TGF-β1, by specifically binding to their receptors, might be involved in the myocardial synthesis of BNP during VA in vivo.

## 1. Introduction

Sudden cardiac death (SCD) is a type of sudden, unexpected death caused by a loss of cardiac function [1]. In Asia, the incidence of SCD is 40/100,000 persons, less than the figure of 50 to 100/100,000 persons reported for North America and Europe [2]. Given that lethal arrhythmias are responsible for most sudden deaths, ventricular arrhythmias (VA), especially ventricular tachycardia (VT) or ventricular fibrillation (VF), are thought responsible for most cases [3]. Since death occurs, sometimes in a matter of minutes or even seconds, the identification of functional indicators that can determine the occurrence of lethal ventricular tachyarrhythmia (LVTA) before SCD is important for the purposes of both determining a clinical diagnosis and in forensic identification [4].

Brain natriuretic peptide (BNP) and endothelin-1 (ET-1) are both crucial biochemical indicators of cardiac dysfunction [5,6,7,8]. Under physiological conditions, only a small amount of BNP is stored in myocardial tissues. Once cardiomyocytes become stretched in response to mechanical strain, such as in pressure overload or volume expansion of the ventricle, ventricular myocytes begin to synthesize and secrete BNP [9]. Clinical studies have shown that cardiomyocytes can synthesize and secrete BNP during VA and that BNP in plasma has a very high predictive value for the occurrence of arrhythmias in patients with congestive heart failure (CHF) [10,11]. ET-1 is a potent coronary vasoconstrictor that plays an active role in arrhythmogenesis [12,13]. Vitro experiments have shown that mechanical stress also acts on cardiomyocytes. The synthesis of BNP induced by mechanical stress on cardiomyocytes is most likely achieved by the mechanical stress-induced synthesis of ET-1 [14,15,16,17]. ET-1 can also lead to myocardial fibrosis by stimulating transforming growth factor-beta 1 (TGF-β1), which plays a vital role in the occurrence and development of cardiac diseases such as cardiomyopathy, heart valve disease, and arrhythmias [18,19,20]. In the hearts of newborn mice, hypoxia can induce TGF-β1 to activate various signaling pathways that act on the promoter of BNP, eventually activating the expression of BNP [21]. Conversely, BNP can inhibit cardiac fibroblasts by blocking TGF-β1/Smad signaling [22]. Coinstantaneous upregulated TGF-β1 and BNP during myocardial remodeling in rats also indicates a possible relationship between these factors [20,22].

To the best of our knowledge, investigations are lacking on the expression patterns of BNP, ET-1, and TGF-β1 in the myocardium during BaCl_2_-induced VA in vivo. For this purpose, the present study investigated the expression of BNP, ET-1, and TGF-β1 proteins and their mRNA in the myocardial tissues of rats with VA. PD142893 (ET-1 receptor blocker) and SB431542 (TGF-β1 receptor type I [TβRI] blocker) were subsequently used to explore the effects of ET-1 and TGF-β1 on BNP, and mechanisms of BNP expression during VA in vivo.

## 2. Results

### 2.1. Electrocardiogram Tracings of Rats Exhibiting VA Induced by BaCl_2_ Solution

**Figure 1:**
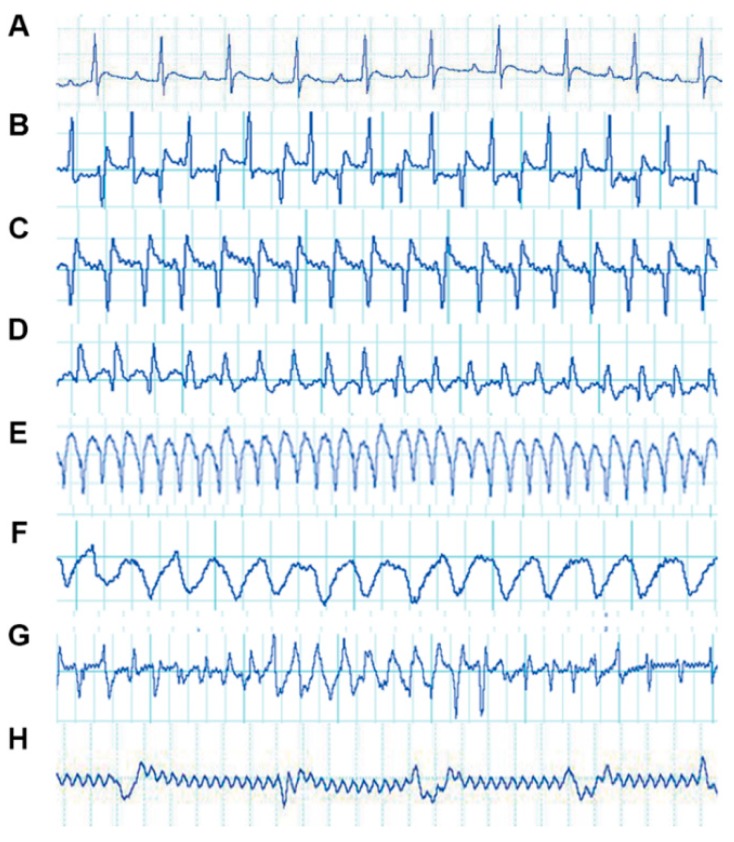
Electrocardiogram (ECG) tracings of rats with ventricular arrhythmias (VA) induced by BaCl_2_. The vertical axis of each ECG indicates voltage (mV), with 0.5 mV for all grids. The horizontal axis of the ECG indicates time (s), with 0.2 s for all four grids: (**A**) normal rat ECG tracing; (**B**) supraventricular tachycardia (SVT); (**C**) ventricular tachycardia (VT); (**D**) myocardial ischemia; (**E**) ventricular flutter; (**F**) ventricular fibrillation (VF); (**G**) different types of arrhythmias switching between each other; and (**H**) a terminal ECG.

The electrocardiogram (ECG) tracings of rats are shown in Figure 1. In the saline group, normal ECGs were recorded (Figure 1A). Rats showed abnormal ECG waveforms 5.26 ± 1.78 s after the injection of BaCl_2_ solution. Waveforms indicating VT and supraventricular tachycardia (SVT) were the first to appear (Figure 1B,C). After a short period of injection, most ECGs of animals showed a return to normal (Figure 1A). When rats underwent repeated injections, ventricular flutter (Figure 1E) and VF (Figure 1F) occurred successively. At the same time, ECGs also displayed waveforms of myocardial ischemia (Figure 1D). Different types of arrhythmias occurred, switching between each other (Figure 1G). All animals died from VF (Figure 1F) after a lethal dose of BaCl_2_. The ECGs of animals prior to their deaths are shown in Figure 1H. ECG tracings indicated that a model of VA induced by BaCl_2_ was successfully established in rats.

### 2.2. Hemodynamic and Histologic Changes in the Left Ventricle of Rats with VA

**Figure 2:**
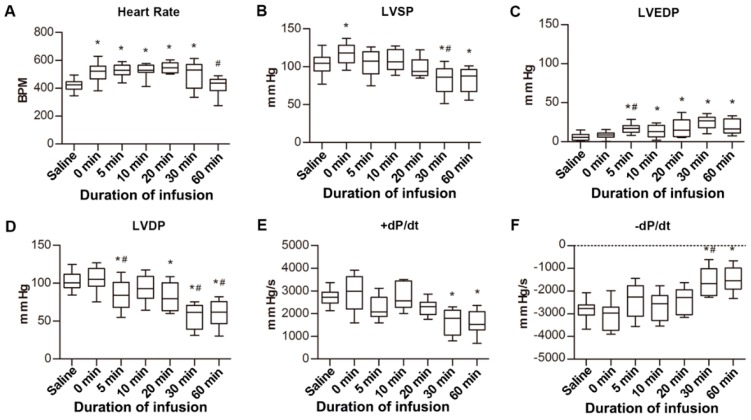
Left ventricular hemodynamic parameters of rats with ventricular arrhythmia (VA). (**A**) Heart rates of rats after injection of BaCl_2_ solution. BPM, beats per minute. (**B**) Left ventricular systolic pressure (LVSP) of rats after injection of BaCl_2_ solution. (**C**) Left ventricular end-diastolic pressure (LVEDP) of rats after injection of BaCl_2_ solution. (**D**) Left ventricular developed pressure (LVDP) of rats after injection of BaCl_2_ solution. (**E**,**F**) +dP/dt and −dP/dt of rats after injection of BaCl_2_ solution. All groups were compared to the saline group. * *p* < 0.05 vs. saline group. # *p* < 0.05 vs. previous timepoint group.

Compared to the saline group, the heart rates of rats with VA increased significantly (*p* < 0.05), except for the 60 min group (Figure 2A). Left ventricular systolic pressure (LVSP) increased immediately as the arrhythmia occurred, but showed declines at 30 min and 60 min (Figure 2B). The left ventricular end-diastolic pressure (LVEDP) increased at 5 min after VA and was maintained for 60 min (Figure 2C). The left ventricular developed pressure (LVDP) decreased continuously (Figure 2D). Compared with the saline group, the +dP/dt decreased 30 and 60 min after VA, while the opposite occurred for −dP/dt (Figure 2E,F).

**Figure 3:**
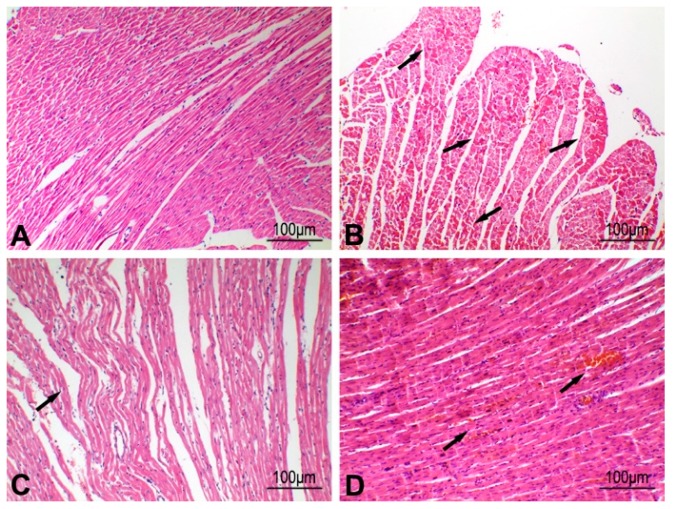
Hematoxylin-eosin (H-E) staining of myocardium after ventricular arrhythmia (VA) in rats. (**A**) Normal left ventricular myocardium of rats. (**B**) The left ventricular myocardium showed enhanced eosinophil staining (arrows) 10 min after VA in rats. (**C**) The left ventricular myocardium showed myocardial wave-like changes (arrow) 30 min after VA in rats. (**D**) The left ventricular myocardium showed myocardial interstitial hemorrhage (arrows) 60 min after VA in rats.

After a period of VA, nonspecific changes, such as enhanced eosinophil staining and myocardial interstitial hemorrhage, were also observed in myocardial tissue after myocardial ischemia was examined by hematoxylin-eosin (H-E) staining (Figure 3). These results suggested that both arrhythmia and myocardial ischemia could occur at the same time, and prolonged VA or myocardial ischemia could result in cardiac dysfunction.

### 2.3. Increased Expression of ET-1 and BNP in Myocardial Tissues after VA

**Figure 4:**
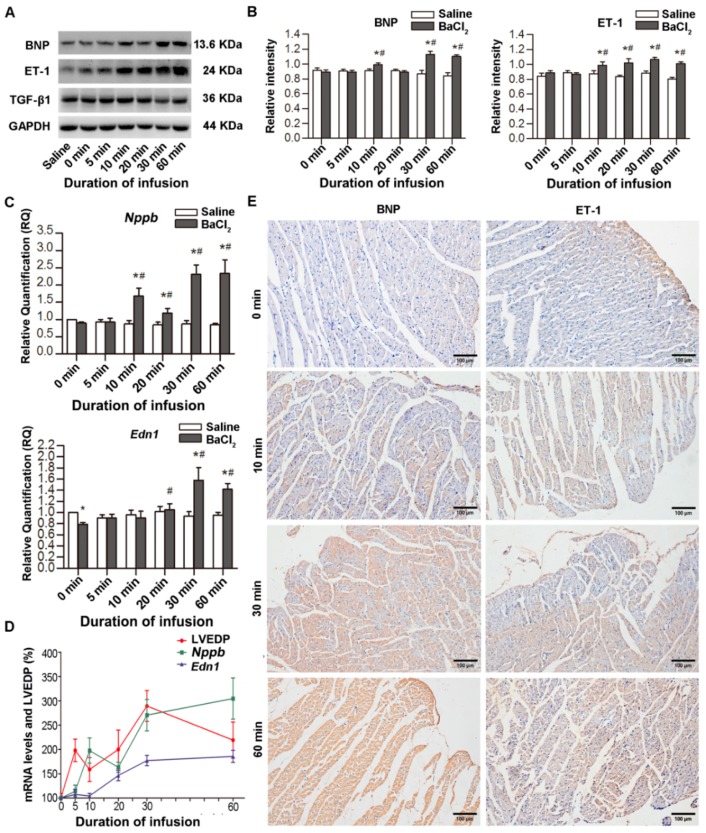
Expression of Endothelin-1 (ET-1), brain natriuretic peptide (BNP), and transforming growth factor-beta 1 (TGF-β1) in myocardial tissues after ventricular arrhythmia (VA). (**A**,**B**) BNP, ET-1, and TGF-β1 protein expression in myocardial tissues of rats after VA by western blot. (**C**) Expression of BNP mRNA (*Nppb*) and ET-1 mRNA (*Edn1*) in left ventricular myocardial tissues of rats after VA by real-time quantitative polymerase chain reaction (qPCR). (**D**) Correlation of left ventricular end-diastolic pressure (LVEDP) with *Nppb* and *Edn1*. The mean value of each indicator in the 0 min subgroup of the BaCl_2_ group was set as 100%. (**E**) Immunohistochemical (IHC) staining for BNP and ET-1 expression in rat myocardium of the BaCl_2_ subgroup. * *p* < 0.05, BaCl_2_ group vs. saline group in the same time subgroup. # *p* < 0.05, different time subgroups vs. 0 min subgroup in the BaCl_2_ group.

The expression of ET-1, BNP and TGF-β1 proteins after VA was assessed by western blotting (Figure 4A,B) and immunohistochemical (IHC) staining (Figure 4E). Glyceraldehyde-3-phosphate dehydrogenase (GAPDH) was used as an internal control for the expression of BNP, ET-1, and TGF-β1 proteins in the rat myocardium. Compared with the saline group, the ratios of BNP, ET-1, and TGF-β1 to GAPDH were almost equal at 0 min. The ratio of BNP to GAPDH began to increase at 10 min, then slightly decreased at 20 min, increased again at 30 min, and lasted for 60 min. The ratio of ET-1 to GAPDH increased at 10 min after VA and lasted for 60 min. Moreover, real-time quantitative polymerase chain reaction (qPCR) further revealed that the expression of *Nppb* (BNP mRNA) and *Edn1* (ET-1 mRNA) genes was closely associated with sustained arrhythmias (Figure 4C). The change in *Nppb* was the same as that of BNP protein; that is, increased after 10 min of VA and increased again after a slight decrease at 20 min. *Edn1* decreased significantly at 0 min and increased significantly after 20 min of VA. TGF-β1 and *Tgfb1* (TGF-β1 mRNA) did not show significant changes (Figure 4A and Figure S1). Considering the association between LVEDP and VA, trends in changes of LVEDP, *Edn1*, and *Nppb* after VA at different time points are plotted in Figure 4D. Within 30 min of VA, *Nppb, Edn1,* and LVEDP showed the same trends in changes with continuous VA compared with each’s previous timepoint: initially increasing, decreasing, and increasing again. However, the reaction time of *Nppb* lagged slightly at 10 min. After 60 min of VA, LVEDP decreased due to decompensated cardiac function, but *Nppb* and *Edn1* kept increasing. Thus, ET-1 and BNP proteins and mRNAs increased with time after VA; however, TGF-β1 protein remained unchanged.

### 2.4. PD142893 and SB431542 Inhibited BNP Expression in the Myocardium

To explore the effect of ET-1 and TGF-β1 on the synthetic mechanism of BNP in the VA model, different concentrations of PD142893 and SB431542 were used in a preliminary experiment. We chose a common solvent (saline, Tween-80, DMSO; 18:1:1) for drug dissolution and this was used for rats of the vehicle group. The expression of BNP in myocardial tissue of rats in the vehicle group did not differ with that of rats in the BaCl_2_ group. We chose PD142893 at a concentration of 0.1 mg/kg and SB431542 at a concentration of 2.5 mg/kg for subgroups of different infusion times based on the results of the preliminary experiment (Figure S2).

**Figure 5:**
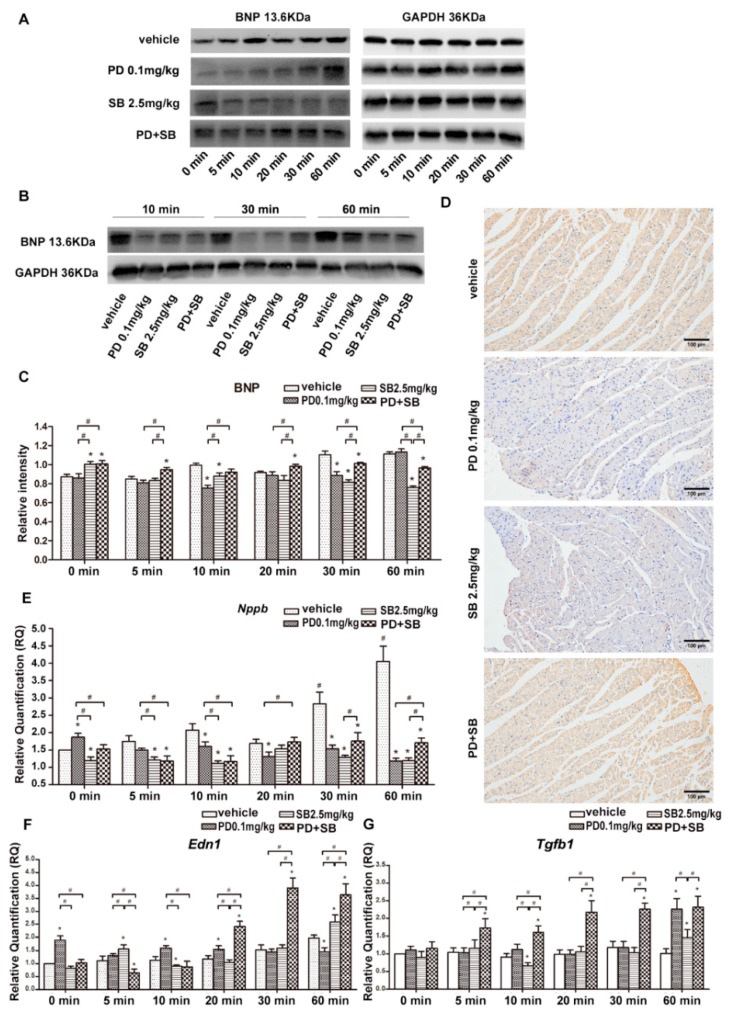
Expression of brain natriuretic peptide (BNP) protein after the application of inhibitors. (**A**,**C**) Western blot and column graphs of BNP protein in myocardial tissues of rats in the vehicle group. PD 0.1 mg/kg, SB 2.5 mg/kg, or PD+SB groups by western blot analysis. (**B**) Western blot of BNP protein in myocardial tissues of rats with ventricular arrhythmia (VA) at 10, 30, and 60 min after intraperitoneal injection of vehicle, PD 0.1 mg/kg, SB 2.5 mg/kg, or PD+SB before VA. (**D**) Immunohistochemical (IHC) staining for BNP in myocardial tissues of rats with VA 30 min after intraperitoneal injection of vehicle, PD 0.1 mg/kg, SB 2.5 mg/kg, or PD+SB before VA. (**E**–**G**) The expression of *Nppb*, *Edn1*, and *Tgfb1* after the application of inhibitors by real-time quantitative polymerase chain reaction (qPCR). PD, PD142893; SB, SB431542. * *p* < 0.05, vs. vehicle group. # *p* < 0.05, vs. same timepoint subgroup.

Compared with the vehicle group, the concentration of BNP protein in the myocardium at 10 and 30 min for rats of the PD 0.1 mg/kg group was significantly decreased. In rats of the SB 2.5 mg/kg group, the BNP protein level increased at 0 min, then decreased at 5 min, and was at a low level until 60 min. The expression of BNP protein in rats of the PD+SB group was markedly high and lasted for 60 min, although it was lower than that of rats in the vehicle group at 30 and 60 min (Figure 5A–C). The IHC staining of BNP after 30 min of VA was consistent with that shown in western blot analysis (Figure 5D). In addition to the 0 min subgroup of PD 0.1 mg/kg, *Nppb* levels were downregulated for PD 0.1 mg/kg and SB 2.5 mg/kg subgroups (Figure 5E). After combining inhibitors, *Nppb* expression at 20, 30, and 60 min was higher than that after a single application of inhibitor, but it was still at a lower level than in the vehicle group (Figure 5E). After the application of PD 0.1 mg/kg, the concentration of *Edn1* in the myocardium increased from 0 to 20 min, while it decreased at 60 min compared with the vehicle group at the same timepoints; after the application of SB 2.5 mg/kg, *Edn1* significantly increased at 60 min after VA (Figure 5F). After the combined application of PD142893 and SB431542, *Edn1* was significantly upregulated at 20 min after VA and lasted for 60 min (Figure 5F). As for *Tgfb1*, it was highly expressed at 5 min, after combining inhibitors, and this lasted for 60 min (Figure 5G). The results indicated that ET-1 and TGF-β1 may be involved in the secretion of BNP in rat myocardial tissues during VA in vivo.

## 3. Discussion

Identifying novel biomarkers for the occurrence of fatal arrhythmias, especially VA, is an important long-standing goal for the clinical diagnosis and forensic identification of SCD [23]. In order to explore the expression of BNP, ET-1, and TGF-β1 in myocardial tissues of rats with VA, we established a VA rat model by injecting a small amount of highly concentrated BaCl_2_ solution. After the stable induction of VA in rats, concentrations of BNP and ET-1 in the myocardium of rats significantly increased over time. In order to investigate, in a preliminary manner, the association between BNP, ET-1, and TGF-β1, two receptor antagonists (PD142893 and SB431542) were used in the rat VA model. We found that after the application of each inhibitor, BNP was significantly downregulated in the myocardium of rats, but could be reversed after the co-application of these two inhibitors. These results suggested ET-1 and TGF-β1 may be involved in the secretion of BNP in vivo during VA, and this may occur by the combined activity of the corresponding receptors.

BNP was originally extracted from porcine brains and was found to have several biological functions including in sodium diuresis, blood pressure vasodilatation, anti-fibrotic activity, and the inhibition of myocardial remodeling [24]. Since it reflects cardiac function, BNP is widely used as a biomarker in the diagnosis of heart failure [25,26,27]. In addition, the secretion of BNP is one of the functions of cardiac self-regulation; it also has a physiological self-protective response, particularly when VA occurs [28]. Levine et al. showed that elevated baseline N-terminal proBNP and BNP levels are independently associated with a risk for ventricular tachyarrhythmias in patients receiving implantable cardioverter-defibrillators for primary intervention purposes [10]. However, whether elevated BNP levels can predict SCD induced by fatal VA remains controversial, although our previous study demonstrated that BNP protein and mRNA were up-regulated in the myocardium and plasma of rats during nonspecific arrhythmias in vivo [29]. In the present study, after the stable induction of VA in rats by intraperitoneal injection of BaCl_2_ solution, BNP protein and mRNA in myocardial tissue significantly increased over time, highlighting a positive correlation between BNP and VA. The earliest differential expression time was 10 min after VA, suggesting BNP is a biomarker for the occurrence of VA in SCDs.

We also found that ET-1 was elevated with a similar pattern to BNP during VA. The expression of ET-1 was consistent with increased BNP during the occurrence of arrhythmias as clinical studies have previously shown [30,31]. ET-1 is the most potent vasoconstrictor in the human cardiovascular system and has remarkably long-lasting actions [32]. It contributes to a reduction in cardiac output, the stimulation of cardiac hypertrophy, and fibroblast proliferation by activating two G-protein–coupled receptors: endothelin receptor type A (ETA) and endothelin receptor type B (ETB) [13]. Moreover, ET-1 is capable of hindering local electrocardio-conduction and thus modulating arrhythmia [12,33,34,35]. Changes in cardiac mechanical stress are the main factors leading to the expression of ET-1, as well as BNP [34,36,37,38,39]. Hemodynamic indexes, such as LVSP, LVEDP, and +dP/dt, reflect the cardiac ejection fraction and therefore indicate cardiac function. Changes in LVEDP are most closely related to pressure overload or volume expansion of the ventricle, which may affect BNP secretion and also reflect cardiac function [40]. In the present study, within 20 min of the occurrence of VA, the heart rates of rats increased due to tachycardia. With an increase in the duration of arrhythmia, although diastolic function was impaired, it was still in a compensatory period. At later stages of VA (30 and 60 min), the cardiac compensatory function was reduced and gradually entered the decompensated stage. The decrease in heart rate at 60 min also indicated abnormal heart function from another aspect. The expression of ET-1 in myocardial tissues of rats after VA was increased slightly ahead of increases in BNP and ET-1 in the myocardial tissue, which is generally consistent with previous findings [30]. In addition, the changing trend shown by *Edn1* within 30 min of VA was exactly the same as that of LVEDP and occurred slightly ahead of that of *Nppb*. These findings indicated that ET-1 may be used as a new indicator of VA in SCD, although this requires further verification by other studies.

In addition, TGF-β1 has been recognized as a key cytokine in the formation and development of cardiac fibrosis since it activates smad signaling [41,42]. It can induce the expression of ET-1 in macrophages, fibroblasts, endothelial cells, and cardiomyocytes, thereby promoting myocardial fibrosis [34]. Researchers also found that hypoxia can induce TGF-β1 to activate the promoter of BNP and eventually activate the expression of BNP in the newborn mouse heart and during myocardial infarction [21]. In rat models with a deleted *Nppb*, myocardial fibrosis occurred when the pressure load was increased, which suggested that there may be some unknown correlation between BNP secretion and TGF-β1 in cardiovascular diseases [22]. Once the TGF-β1/Smad signaling pathway is inhibited, BNP can reduce hypertrophy and ventricular remodeling of cardiac myocytes after heart failure [15,43,44]. However, we did not observe an elevation in TGF-β1 during VA, which may be due to the short-term VA induced.

Furthermore, no previous study has observed the effect of ET-1 and TGF-β1 on expression patterns of BNP during VA in vivo. For this reason, we used PD142893 (ET-1 receptor blocker) and SB431542 (TGF-β1 receptor type I blocker) to investigate their influences on the myocardial expression of BNP during VA. PD142893 can non-selectively antagonize ETA/B receptors, thus inhibiting ET-1 binding to ETA/B receptors and hindering ET-1 physiological function, and SB431542 is a small-molecule inhibitor of TβRI [45,46]. After treatment with PD142893 and SB431542 in suitable concentrations, the expression of BNP was significantly decreased. Interestingly, after the combined application of PD142893 and SB431542, although the expression of *Nppb* was lower than that of the vehicle group, BNP protein showed a persistently high expression. The results suggested that ET-1 and TGF-β1 may be involved in the secretion of BNP in rat myocardial tissues during VA in vivo; any regulatory function may be realized by the combined activity of corresponding receptors. The reversal after the co-application of inhibitors indicated that in the occurrence of VA—except for ET-1 and TGF-β1, which were revealed in this experiment to be involved in the generation of BNP—other unknown regulatory pathways enhance the observed positive regulatory effect of inhibitors on BNP; however, the specific mechanism involved needs to be further studied. Furthermore, *Tgfb1* was significantly upregulated after the combined application of the two inhibitors. We speculate that once the synthesis of BNP has been significantly inhibited, the small amount of BNP remaining cannot protect the heart adequately, therefore promoting the expression of *Tgfb1*.

Several limitations exist in this study. One limitation is that although we can induce VA in rats by BaCl_2_, it is inevitable that atrial or supraventricular arrhythmias will last for 1 h in the process of VA. Therefore, animals dominated by non-ventricular arrhythmias were not included in the study. Secondly, although inhibitors were commonly used in exploring regulatory mechanisms, these were mainly used in in vitro experiments. Unfortunately, as arrhythmia is a complex pathological process, it cannot be simulated in vitro with the current techniques; thus, we chose intraperitoneal injection to explore the initial phenomenon. Since the two inhibitors are specific receptor blockers, we conducted preliminary experiments to ensure the drugs worked. Thirdly, as mentioned above, the synthesis and the secretion of BNP are regulated by a variety of neurohumoral factors. In the current study, we aimed to explore the effect of ET-1 and TGF-β1 on BNP in vivo. We will continue to study the effects of other factors caused by complex regulatory networks. Considering the complex pro-fibrotic role of TGF-β1 together with BNP and ET-1, the expression levels of these three in other cardiac diseases are also worthy of investigation.

## 4. Materials and Methods

Data, analytic methods, and study materials will be/have been made available to other researchers for the purposes of reproducing the results or replicating procedures.

### 4.1. Animals and Induction of Cardiac Arrhythmias by BaCl_2_

All experiments performed in the present study strictly conformed to the “Guide for the Care and Use of Laboratory Animals” prepared by the Institute of Laboratory Animal Research and published by the National Institutes of Health (NIH Publication No. 86–23, Revised 1985). These sought to minimize both the number of animals used and any suffering that they might experience. The experiments were also carried out according to the Guidelines for the Care and Use of Laboratory Animals of China Medical University.

Sprague-Dawley (SD) male rats (from the Animal Center of China Medical University) weighing 300–350 g were kept at a controlled room temperature under a 6 a.m. to 6 p.m. light regime and fed regular pelleted rat chow and tap water *ad libitum*. The animal model was established based on previous studies [47]. In brief, rats were placed in a supine position throughout experiments after being anesthetized by intraperitoneal injection with 2% sodium pentobarbital (1.5 mg/100 g body weight). A solution of 10% BaCl_2_ (10 mg/100 g body weight) was injected intravenously by a microinjector pump at a dose of 0.004 mL/100 g body weight to establish the animal model. A Powerlab biological signal processing system (AD Instrument, Bella Vista, Australia) was used to record ECG signals from Lead II, heart rates, and hemodynamic changes.

First, SD male rats (n = 72) were divided into BaCl_2_ and saline groups (each n = 36). BaCl_2_ group animals were injected with BaCl_2_ and killed by a double dose of BaCl_2_ at the corresponding timepoint (0, 5, 10, 20, 30, 60 min), while saline group animals were injected with the same volume of saline and sacrificed by dislocation of the cervical vertebra. BNP, ET-1, and TGF-β1 proteins and mRNA were detected in the myocardium of rats by IHC staining, western blot, and real-time qPCR, respectively. After determining the appropriate inhibitor concentration, SD male rats (n = 144) were randomly divided into four groups (each n = 36): vehicle, PD (0.1 mg/kg), SB (2.5 mg/kg), and PD + SB. The corresponding inhibitor solution was intraperitoneally injected 30 min before the induction of arrhythmia. Each group of rats was sacrificed as six sub-groups (each n = 6) according to a previously used protocol. The amount of BNP generated was compared after animals were killed.

### 4.2. Western Blot

Myocardium specimens were washed in phosphate buffered saline (PBS) and lysed with radioimmunoprecipitation buffer (sc-24948, Santa Cruz Biotechnology, Santa Cruz, CA, USA) containing 10 μg/mL of the protease inhibitor, phenylmethylsulfonyl fluoride. After centrifugation (12,000× *g*, 10 min, 4 °C) three times, the amount of protein in the supernatant was measured using bovine serum albumin (SK3051, Sangon Biotech, Shanghai, China) as a standard. Proteins were then subjected to sodium dodecyl sulfate–polyacrylamide gel electrophoresis followed by western blotting. Antigen–antibody complexes were detected by antibodies: anti-BNP diluted 1:500 (AB1549; Millipore, Burlington, MA, USA), anti–ET-1 diluted 1:1000 (ab2786; Abcam, Cambridge, UK), TGF-β1 diluted 1:2000 (ab92486, Abcam), and anti-GAPDH diluted 1:5000 (ab8245, Abcam) at 4 °C overnight. Horseradish peroxidase-labelled goat anti-rabbit IgG (ZB-2301; Zhongshan Golden Bridge Biotechnology, Beijing, China) or rabbit anti-goat IgG (ZB-2306, Zhongshan Golden Bridge Biotechnology) was used at a 1:5000 dilution at room temperature for 2 h. An enhanced chemiluminescence detection system, Electrophoresis Gel Imaging Analysis System (MFChemiBIS 3.2; DNR Bio-Imaging Systems, Jerusalem, Israel), was used to visualize blots.

### 4.3. H-E and IHC Staining

Specimens of cardiac tissues were fixed in 4% paraformaldehyde and then embedded in paraffin. Samples were dehydrated in gradient alcohol and pellucidum in xylene. Each paraffin block was then cut into 5-μm thick sections and mounted onto slides with 3-aminopropyl-triethoxysilane. Sections were identified using H-E staining at a 200-fold magnification by light microscopy. Immunostaining was performed using streptavidin peroxidase and diaminobenzidine according to the manufacturer’s instructions (Zymed Laboratories, South San Francisco, CA, USA). In brief, conventionally made 5-μm thick sections of cardiac tissue were heated for 10 min in 0.01 mol/L sodium citrate buffer (pH 6.0) with a microwave oven for antigen retrieval. Subsequently, 3% hydrogen peroxide was added to quench endogenous peroxidase activity. The sections were then blocked with 10% nonimmune goat serum to reduce nonspecific binding and incubated with antibodies: anti-BNP diluted 1:500 (AB1549, Millipore), anti–ET-1 diluted 1:500 (ab2786, Abcam), and anti–TGF-β1 diluted 1:200 (ab92486; Abcam) overnight at 4 °C. After washing in PBS, horseradish peroxidase-conjugated secondary antibody was added to sections and these incubated with diaminobenzidine tetrahydrochloride. The sections were then mounted with gummi, after staining the nucleus with hematoxylin, and dehydrated in gradient alcohol and pellucidum in xylene.

### 4.4. Quantitative Real-Time Quantification Polymerase Chain Reaction

Each 100 mg of myocardium specimen was ground after being frozen in liquid nitrogen. Total RNA was extracted with RNAiso Plus (D9108; Takara Biotechnology, Shiga, Japan) and immediately reverse-transcribed into cDNA with a PrimeScript RT reagent Kit (DRR037, Takara). Aliquots of 10 µL of reaction mixture containing 2 µL of PrimeScript Buffer (5 ×), 0.5 µL PrimeScript RT Enzyme Mix I, 0.5 µL Oligo Dt Primer (50 µM), 0.5 µL Random 6 mers (100 µM), 4.5 μL ddH_2_O, and 2 µL total RNA (400 ng) with GeneAmp 9700 (Life Technologies, Carlsbad, CA, USA) were used under the following condition: 37 °C for 15 min, 85 °C for 5 s, and 4 °C for 5 min. Real-time qPCR was performed with SYBR Premix Ex Taq II (TliRNaseH Plus; RR820A, Takara) with a 7500 real-time PCR system (Life Technologies) in triplicate. Relative quantification was carried out by a comparative CT (ΔΔCT) method to simultaneously test the cDNA of BNP, ET-1, TGF-β1, and the endogenous reference (GAPDH). Aliquots of 20 µL of the reaction mixture containing 6 µL ddH_2_O, 10 µL SYBR Premix Ex Taq II (2 ×), 0.8 µL PCR forward primer (10 µM), 0.8 µL PCR reverse primer (10 µL), 0.4 µL ROX Reference Dye II (50 ×), and 2 µL cDNA were used under the following amplification conditions: 95 °C for 30 s, 40 cycles at 95 °C for 5 s, and 60 °C for 34 s. Primers for BNP, ET-1, TGF-β1,and GAPDH, designed by Web-based IDT SciTools real-time PCR software, are listed in Table 1.

### 4.5. Statistical Analysis

All data are represented as the mean ± standard deviation (SD). Differences among subgroups with different treatments, which were normally distributed variables, were analyzed by one-way analysis of variance (ANOVA); post hoc analyses were performed using Dunn’s test. The difference between the two different treatments was analyzed by unpaired *t*-test. All statistical processing was performed using PRISM 6.0 software. *p* < 0.05 was considered to be statistically significant.

## 5. Conclusions

The expression of BNP and ET-1 protein and mRNA increased after BaCl_2_-induced VA and was associated with the duration of VA, while TGF-β1 protein did not change. We found that ET-1 and TGF-β1 may be involved in the synthesis of BNP in the myocardium during VA, and that the mechanism of action may be through specific binding of their respective receptors.

## Figures and Tables

**Table 1:** Primer sequences used for reverse transcription PCR.

Gene	Species	mRNA	Primer
*Nppb*	Rattus norvegicus	NM_031545	Forward: 5′-CTTTTCCTTAATCTGTCGCCG-3′
			Reverse: 5′-GTCTCTGAGCCATTTCCTCTG-3′
*Edn1*	Rattus norvegicus	NM_012548	Forward: 5′-TGGTGGAGGGAAGAAAACTAAG-3′
			Reverse: 5′-CTCTGTAGTCAATGTGCTCGG-3′
*Tgfb1*	Rattus norvegicus	NM_021578	Forward: 5′-CCTGAGTGGCTGTCTTTTGA-3′
			Reverse: 5′-CGTGGAGTACATTATCTTTGCTG-3′
*Gapdh*	Rattus norvegicus	NM_017008	Forward: 5′-TCCAGTATGACTCTACCCACG-3′
			Reverse: 5′-CACGACATACTCAGCACCAG-3′

## Supplementary Materials

Supplementary materials can be found at https://www.mdpi.com/1422-0067/20/23/5845/s1.

## Abbreviations

VA	Ventricular arrhythmia
SCD	Sudden cardiac death
BNP	Brain natriuretic peptide
ET-1	Endothelin-1
TGF-β1	Transforming growth factor beta 1
IHC	Immunohistochemistry
qPCR	Quantitative polymerase chain reaction
*Nppb*	BNP mRNA
*Edn1*	ET-1 mRNA
*Tgfb1*	TGF-β1 mRNA
VT	Ventricular tachycardia
VF	Ventricular fibrillation
ECG	Electrocardiogram
LVSP	Left ventricular systolic pressure
LVEDP	Left ventricular end-diastolic pressure
LVDP	Left ventricular developed pressure
GAPDH	Glyceraldehyde-3-phosphate dehydrogenase
ETA	Endothelin receptor type A
ETB	Endothelin receptor type B
cDNA	Complementary DNA
