# Supplementary material for: The Expression of BNP, ET-1, and TGF-β1 in Myocardium of Rats with Ventricular Arrhythmias
Source: Int J Mol Sci. 2019 Nov 21;20(23):5845. doi: 10.3390/ijms20235845 (PMC6928624; doi:10.3390/ijms20235845)
Supplement: Supplementary file 1 [file ijms-20-05845-s001.zip › ijms-630058-final-supp/SUPP.docx]

**Supplementary Materials:** The following are available online at www.mdpi.com/xxx/s1, Figure S1: Expression of transforming growth factor (TGF)-β1 protein and mRNA (*Tgfb1*) in myocardial tissues after ventricular arrhythmia (VA). Figure S2. Expression of brain natriuretic peptide (BNP) protein and mRNA (*Nppb*) after the application of different concentrations of inhibitors.

**
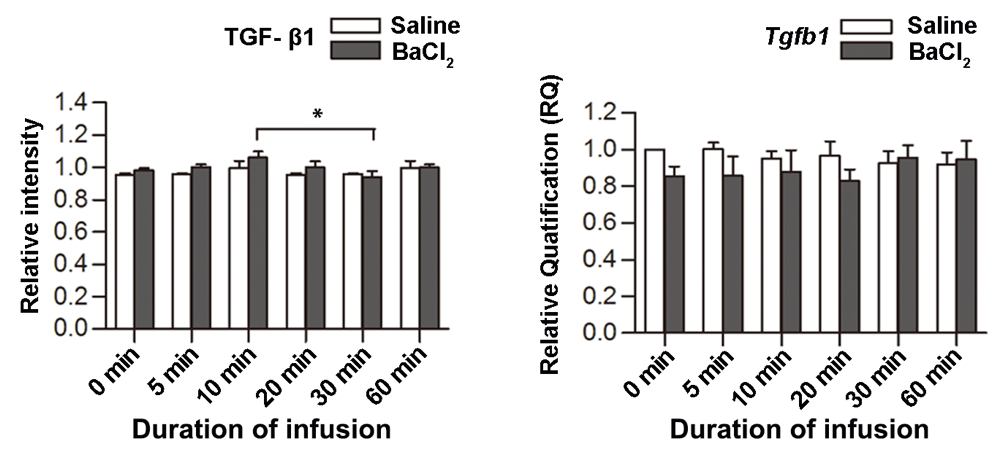
**

**Figure S1.** Expression of transforming growth factor-beta 1 (TGF-β1) protein and mRNA (*Tgfb1*) in myocardial tissue after ventricular arrhythmia (VA). * *p* < 0.05, 10 min vs. 30 min.

*
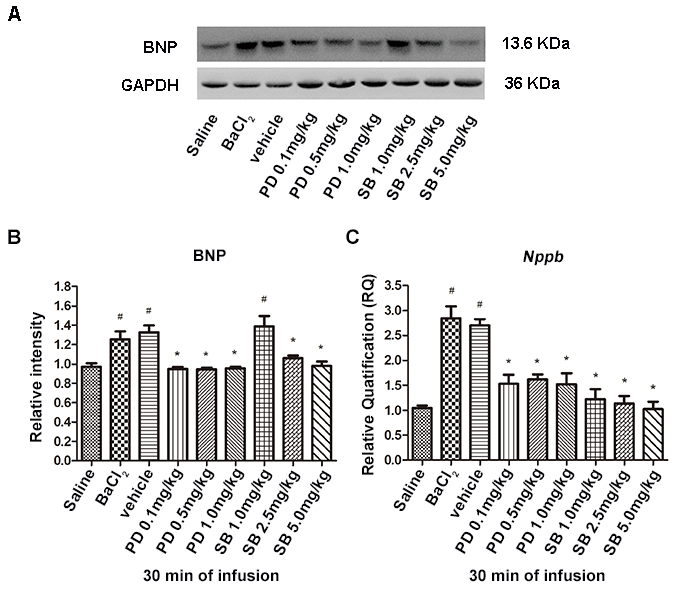
*

**Figure S2.** Expression levels of brain natriuretic peptide (BNP) protein and mRNA (*Nppb*) after the application of different concentrations of inhibitors. (**A**,**B**) Western blot analysis of BNP protein in rat myocardial tissue from different groups. The solvent (saline, Tween-80, DMSO; 18:1:1) for drug dissolution was used in vehicle group animals. (**C**) Histogram of *Nppb* expression in different groups by real-time quantitative polymerase chain reaction (qPCR). PD, PD142893; SB, SB431542. **p* < 0.05, vs. vehicle group. #*p* < 0.05, vs. saline group.
