# Supplementary figures and images for: The Expression of BNP, ET-1, and TGF-β1 in Myocardium of Rats with Ventricular Arrhythmias
Source: Int J Mol Sci. 2019 Nov 21;20(23):5845. doi: 10.3390/ijms20235845 (PMC6928624; doi:10.3390/ijms20235845)

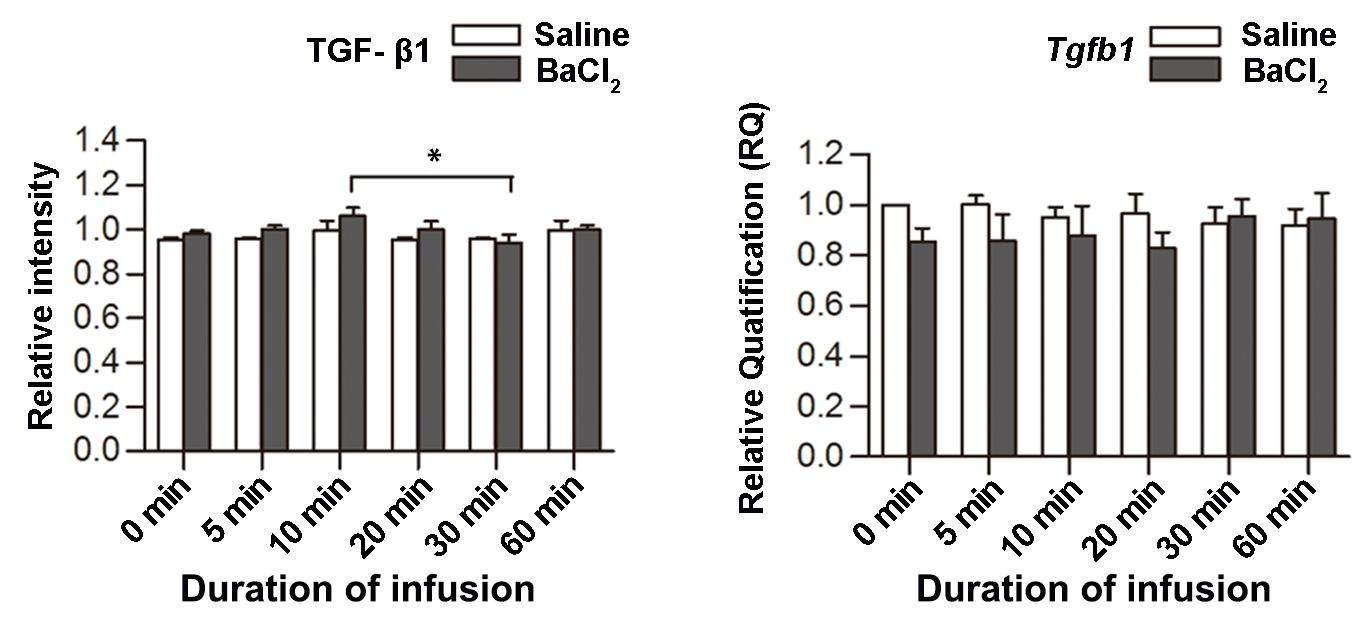

Supplement: Supplementary file 1 [file ijms-20-05845-s001.zip › ijms-630058-final-supp/Supplementary Figures/Figure S1.tif]

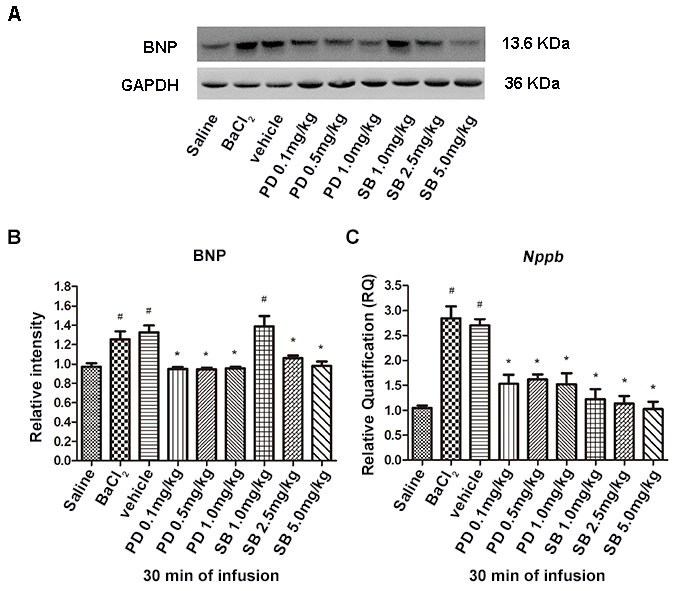

Supplement: Supplementary file 1 [file ijms-20-05845-s001.zip › ijms-630058-final-supp/Supplementary Figures/Figure S2.tif]
